# Prenylflavonoid Isoxanthohumol Sensitizes MCF-7/ADR Cells to Doxorubicin Cytotoxicity via Acting as a Substrate of ABCB1

**DOI:** 10.3390/toxins9070208

**Published:** 2017-06-30

**Authors:** Ming Liu, Weiyi Zhang, Wei Zhang, Xin Zhou, Ming Li, Jinlai Miao

**Affiliations:** 1 Key Laboratory of Marine Drugs, Ministry of Education, School of Medicine and Pharmacy, Ocean University of China, Qingdao 266003, China; 13070868219@163.com (W.Z.); yier542789541@126.com (X.Z.); lmsnouc@ouc.edu.cn (M.L.); 2 Laboratory for Marine Drugs and Bioproducts of Qingdao National Laboratory for Marine Science and Technology, Qingdao 266237, China; 3 Southern Research Institute, 2000 9th Avenue South, Birmingham, AL 35205, USA; wzhang@southernresearch.org; 4 Key Laboratory of Marine Bioactive Substance, The First Institute of Oceanography, State Oceanic Administration, Qingdao 266061, China

**Keywords:** isoxanthohumol, doxorubicin resistance, synergism, ABCB1

## Abstract

Isoxanthohumol is a unique prenylflavonoid with the highest content in beer. Isoxanthohumol has multiple bioactivities and has recently received considerable attention in the scientific community. Nonetheless; its effect on drug resistant cancer cells has rarely been studied. In this paper; we investigated the synergistic effect of isoxanthohumol and doxorubicin on doxorubicin resistant MCF-7/ADR cells. Our results showed that isoxanthohumol sensitized the cytotoxic effect of doxorubicin on MCF-7/ADR cells via increased proliferation inhibition and apoptosis stimulation. Molecular mechanism studies further demonstrated that isoxanthohumol inhibited ABCB1-mediated doxorubicin efflux; stimulated the ATPase activity of ABCB1 (ATP-binding cassette sub-family B member 1); and acted as an ABCB1 substrate. Molecular docking results suggested that isoxanthohumol bound to the central transmembrane domain of ABCB1 and its binding site overlapped with the doxorubicin binding site. The present studies demonstrated that isoxanthohumol was a competitive ABCB1 inhibitor which reversed ABCB1-mediated doxorubicin resistance in MCF-7/ADR cells; and therefore could be further developed to help with overcoming ABCB1-mediated drug resistance.

## 1. Introduction

In addition to radiation therapy and tumor resection, chemotherapy, for example, doxorubicin (DOX) treatment, still plays a major role in breast cancer treatment. However, the clinical efficacy of DOX is challenged by a major obstacle represented by the development of multidrug resistance (MDR) [1]. In the case of DOX treatment in breast cancer, MDR reduces intracellular drug accumulation by activating the efflux of chemotherapeutic agents through membrane transporters, such as the ATP-binding cassette (ABC) proteins, which mainly include p-glycoprotein (Pgp, MDR1, ABCB1 (ATP-binding cassette sub-family B member 1)), the multidrug resistance protein 1 (MRP1, ABCC1 (ATP-binding cassette sub-family C member 1)), and the breast cancer resistance proteins (BCRP, ABCG2 (ATP-binding cassette sub-family G member 2)) [2]. Inhibition of ABC transporters has proven effective in reversing MDR. Dietary and non-dietary phytochemicals have a close relationship with cancer, and this topic has been discussed recently [3]. Much effort has been devoted to searching for phytochemicals that can inhibit ABC transporters and have better safety profiles in the treatment of cancer [4].

**Figure 1:**
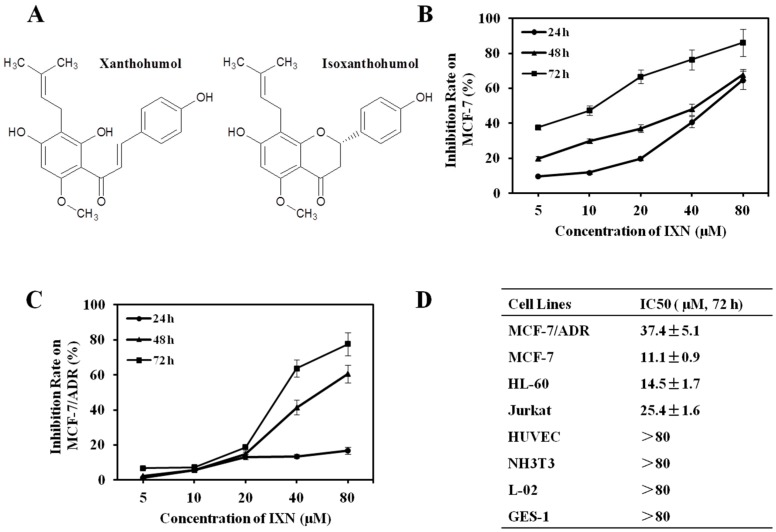
(**A**) Chemical structures of isoxanthohumol (IXN) and xanthohumol (XN). (**B**) IXN inhibits the viability of MCF-7 (**C**) and MCF-7/ADR cells in a concentration- and time-dependent manner. Cells were treated with indicated concentrations of IXN for 24, 48, and 72 h, respectively, and then tested by 3-(4,5)-dimethylthiahiazo(-z-y1)-3,5-di-phenytetrazoliumromide (MTT) assay; (**D**) IC_50_ values of IXN against the indicated cell lines in vitro after treated for 72 h. Data represent means ± SD from three independent experiments.

Prenylflavonoid isoxanthohumol (IXN, Figure 1A) exists in hops *Humulus lupulus* L. and shrub *Sophora flavescens* L. The general dietary source of prenylflavonoid is beer and IXN is the most abundant prenylflavonoid in beer. In beer, IXN is mainly produced from xanthohumol (XN, Figure 1A) thermal isomerization during wort boiling [5]. IXN has attracted considerable attention due to its plant origin, pharmacological properties [5,6], and safety profile. Although not as widely studied as XN, IXN has also been shown to inhibit the proliferation of many kinds of cancer cells [6,7,8], including breast cancer MCF-7 cells [8], and modulate cancer related angiogenesis and inflammation [9]. Interestingly, men consuming beer have a reduced risk of prostate cancer, as compared to those who do not drink beer [10]. IXN also enhances the paclitaxel activity in vivo against melanoma cells [11]. Recent studies revealed that IXN acted as a substrate and inhibitor of the efflux transporter ABCG2 [12], and its isomer XN has also been reported to sensitize MCF-7/ADR cells to DOX cytotoxicity [13]. Considering the high structural similarity between XN and IXN molecules, it would be interesting for finding out whether IXN has the potential to reverse efflux transporter mediated MDR and sensitize MCF-7/ADR cells to DOX, which have not been investigated yet.

In this study, we revealed IXN could enhance the efficiency of DOX on MCF-7/ADR cells by competitively inhibiting the ABCB1 transporting function and increasing the intracellular content of DOX. Our mechanism studies further demonstrated that IXN was an ABCB1 substrate which bound to the same transporter site as DOX.

## 2. Materials and Methods

### 2.1. Materials and Cells

IXN (purity *>* 98%) was provided by School of Medicine and Pharmacy, Ocean University of China. Antibodies against ATP-binding cassette sub-family B member 1 (ABCB1) were purchased from Santa Cruz Biotechnology, Inc., Delaware, CA, USA. Antibodies against cleaved PARP, Mcl-1, Survivin, cleaved caspase 3, cleaved caspase 9, p-ERK, ERK, p-AKT, AKT, p-Stat3, and Stat3 were purchased from Cell Signaling Technology Inc., Danvers, MA, USA. Other reagents and kits were the products of Beyotime Biotechnology, Shanghai, China.

Human breast cancer MCF-7 cells, human promyelocytic leukemia HL-60 cells, human acute T lymphocytic leukemia Jurkat cells, human umbilical vein endothelial cells (HUVECs), mouse fibroblast cell line NIH3T3, human liver cell line L-02, and human gastric epithelium GES-1 cells were provided by the Cell Bank of Chinese Academy of Sciences, Shanghai, China. DOX resistant sub-line MCF-7/ADR was established by a stepwise increase of DOX concentrations in the culture from the parental human breast cancer cell line MCF-7 and maintained in the presence of 0.5 μM DOX as described by Fairchild et al. [14]. MCF-7 were cultured in improved minimum essential medium (MEM), NIH3T3 cells were cultured in Dulbecco’s Modified Eagle Medium (DMEM), and other cells were cultured in RPMI-1640 medium (GIBCO, Grand Island, NY, USA), all supplemented with 10% fetal bovine serum (FBS), penicillin, and streptomycin at 37 °C in 5% CO_2_.

### 2.2. Cell Viability Assessment

The 3-(4,5)-dimethylthiahiazo(-z-y1)-3,5-di-phenytetrazoliumromide (MTT) method was used to assay the cytotoxicity of IXN. Generally, cells were incubated with IXN for different periods. After the MTT solution was incubated for another 4 h, DMSO was added to dissolve the dye crystals. Absorbance was measured at 490 nm.

### 2.3. Calculation of Drug Combination and Synergism

MCF-7/ADR and its parent line MCF-7 cells were treated with DOX and IXN, alone or in combination. After 72 h treatment, MTT assays were performed. The synergistic effect of multiple drugs was analyzed by the definition of Chou and Talalay [15]. The combination index (CI) was calculated using software Calcusyn (Biosoft, Cambridge, UK). CI values of <1, 1, and >1 indicate synergistic, additive, and antagonistic effects, respectively.

### 2.4. Intracellular DOX and Rhodamine123 (Rho123) Accumulation Detection

For the DOX accumulation assay, cells were treated with 3 μM DOX alone or in the presence of IXN (0–30 μM) for 24 h. Cells were harvested, washed, and finally analyzed by themoflo XDP flow cytometer (Beckman-Coulter, Fullerton, CA, USA). The autofluoresence of DOX was excited at 488 nm and detected on the channel of FL2. For the efflux assay of Rho123, cells were pretreated IXN (0–30 μM) for 6 h, followed by incubation with Rho123 (10 μM) for another 2 h at 37 °C. The harvested samples were washed with ice-cold PBS and analyzed by flow cytometry. Excitation and emission wavelengths were 488 and 530 nm for Rho123, respectively.

### 2.5. Western Blotting Assay

After compounds treatments, MCF-7/ADR cells were lysed and total proteins were obtained. Then, the protein samples were separated on SDS-polyacrilamide gels, transferred to nitrocellulose membranes, and probed with primary antibodies and secondary antibodies. FluorChem E (ProteinSimple, San Jose, CA, USA) was used to detect the bands corresponding to the antibodies.

### 2.6. ABCB1 ATPase Activity Assay

The ABCB1 ATPase activity was assayed using Pgp-Glo™ assay systems kit (Promega, Madison, WI, USA). The effects in the presence of IXN and positive control verapamil were compared against the basal ABCB1 ATPase activity. IXN (0–30 μM) or verapamil (200 μM) were incubated with recombinant human ABCB1 membranes (25 μg) in the presence of 5 mM MgATP at 37 °C for 40 min. Then, the remaining ATP was detected according to the manufacture’s protocol, using spectraMax M5 (Molecular Devices, Sunnyvale, CA, USA).

### 2.7. Molecule Docking

Structural model generation and molecular docking studies were conducted using the programs of the Schrödinger Suite 2015 (Schrödinger, LLC, New York, NY, USA, 2015). Two human ABCB1 homology models with different conformations were constructed based on the crystal structures of mouse ABCB1a (PDB IDs:4Q9I, 4Q9L) [16] using the Prime program. The 3D structures of IXN and DOX was prepared using the LigPrep program [17]. The central site at the transmembrane domain (TMD) of ABCB1 was adopted from the ligand binding site of the crystal structures. The ATP binding site at the nucleotide binding domain (NBD) of the ABCB1 model was identified using the SiteMap program. The Glide program was used for docking studies. Specifically, the induced-fit-docking (IFD) protocol [18], which is capable of sampling dramatic side-chain conformational changes as well as minor changes in protein backbone structure, was applied to explore ligand binding modes at different binding sites. IXN and DOX molecules were docked separately to the central pocket as well as the ATP binding site of the ABCB1 homology models. Residues within 5 Å of a docked compound were allowed to be flexible and the docked results were scored using the extra-precision (XP) mode of Glide.

### 2.8. Data Analysis

One-way ANOVA with Tukey’s post hoc test was used for the statistical analysis of the data. The results were expressed as mean values ± SD. Differences of *p* < 0.05 were considered statistically significant.

## 3. Results

### 3.1. IXN Exhibited More Potent Cytotoxicity in MCF-7 Than in MCF-7/ADR Cells

To evaluate the cytotoxicity of IXN, we examined its inhibition effect on cell proliferation. IXN significantly inhibited the growth of DOX sensitive MCF-7 cells in a concentration and time dependent-manner (Figure 1B). As a comparison, the DOX resistant MCF-7/ADR cells were relatively less sensitive to IXN especially at low concentrations (5–20 μM) of IXN (Figure 1C). The IC_50_ values resulted based on 72 h IXN treatment against MCF-7 and MCF-7/ADR cells were 11.1 ± 0.9 and 37.4 ± 5.1 μM, respectively (Figure 1D). IXN also showed similar inhibitory effects on suspension cells HL-60 and Jurkat cells but had minimum effects on different control normal cell lines (HUVEC, NIH3T3, L-02, and GES-1), which clearly indicated its low cytotoxicity against normal cells (Figure 1D).

### 3.2. Synergic Effect of IXN in Combination with DOX

**Figure 2:**
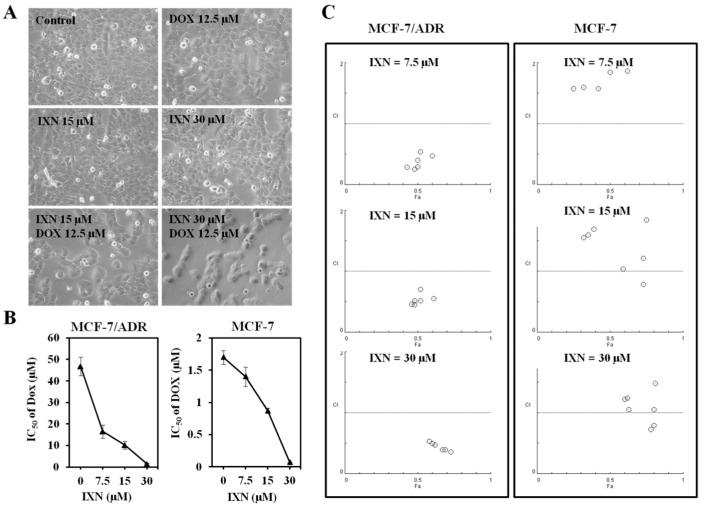
IXN sensitizes the cytotoxicity of doxorubicin (DOX) in MCF-7/ADR cells. (**A**) Representative pictures showing the effect on MCF-7/ADR cell density of DOX alone (12.5 μM), IXN alone (15 and 30 μM), or in combination. (**B**) MCF-7/ADR and MCF-7 cells were treated with DOX in the absence and presence of IXN (7.5, 15, and 30 μM) for 72 h, cell viability was assayed by MTT method and the IC_50_ values of DOX were calculated; (**C**) Median effect plot analysis of synergistic effects. MCF-7/ADR and MCF-7 cells were treated with DOX in the presence of IXN (7.5, 15, and 30 μM) for 72 h, and the combination index (CI) values were calculated by Calcusyn. CI values of <1, 1, and >1 indicate synergistic, additive, and antagonistic effects, respectively.

**Figure 3:**
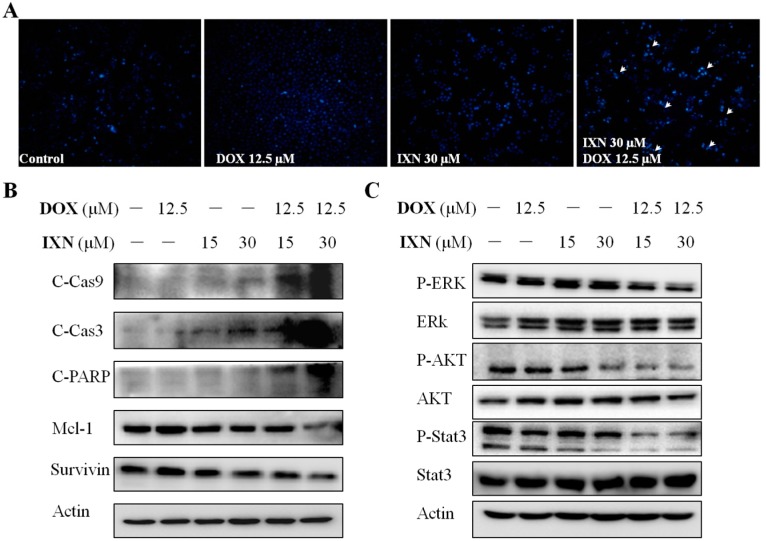
IXN increases DOX-induced apoptosis in MCF-7/ADR cells. Cells were treated with IXN (0–30 μM) alone, DOX (12.5 μM) alone, or in combination for 24 h. (**A**) Cells were stained by Hoechst 33342, and observed by fluorescence micrograph. Shown is a representative result of three separate experiments. (**B**) The apoptosis related molecules and survival related signaling molecules were detected by western blotting.

We then tested whether IXN could reverse the DOX resistance in MCF-7/ADR cells. DOX and IXN alone showed no significant inhibition on the cell density, whereas IXN in combination with DOX significantly decreased the cell population of MCF-7/ADR cells (Figure 2A). The IC_50_ value of DOX on MCF-7/ADR cells was 46.78 ± 4.30 μM. In the presence of IXN at the concentration of 7.5, 15, and 30 μM, the DOX IC_50_ values decreased to 16.49 ± 3.12, 10.16 ± 1.17, and 1.35 ± 0.33 μM, respectively (Figure 3B, left panel). We further conducted an isobolographic analysis to evaluate the synergic effect of DOX and IXN. As shown in Figure 3C (left panel), when DOX combined with IXN at 7.5, 15, and 30 μM, all the CI values were smaller than 1, suggesting strong synergistic behavior between IXN and DOX. Interestingly, when in combination with DOX in DOX-sensitive MCF-7 cells, IXN also decreased DOX IC_50_ values from 1.72 ± 0.11 to 1.40 ± 0.15, 0.87 ± 0.04, and 0.07 ± 0.00 μM (Figure 3B, right panel), respectively. Nonetheless, most of the CI values were larger than 1 (Figure 3C, right panel), suggesting no synergistic effect in MCF-7 cells. Therefore, we only used MCF-7/ADR cells in the following experiments.

### 3.3. Combination of IXN and DOX Stimulated More Apoptosis in MCF-7/ADR Cells

To explore whether IXN in combination with DOX could stimulate more apoptosis in MCF-7/ADR cells, we monitored the morphological changes of nuclear chromatins after Hoechst 33342 staining. As shown in Figure 3A, the number of cell nuclei that exhibited brighter blue fluorescence increased significantly after exposure to IXN and DOX simultaneously, indicating the increased induction of apoptosis with the combined administration of IXN and DOX. The increased apoptosis was further confirmed by the increased level of apoptotic markers, including cleaved caspase 9, cleaved caspase 3, and cleaved PARP, and also the decreased level of anti-apoptosis proteins, such as Mcl-1 and Survivin (Figure 3B). Furthermore, the combination also inhibited the activation of several signaling molecules that hinder cell apoptosis, including p-ERK1/2, p-AKT, and p-Stat3 (Figure 3C). These data demonstrated that IXN could enhance the DOX efficiency in MCF-7/ADR cells by stimulating more apoptosis.

### 3.4. IXN Inhited the Transporting Functions of ABCB1 in MCF-7/ADR Cells

**Figure 4:**
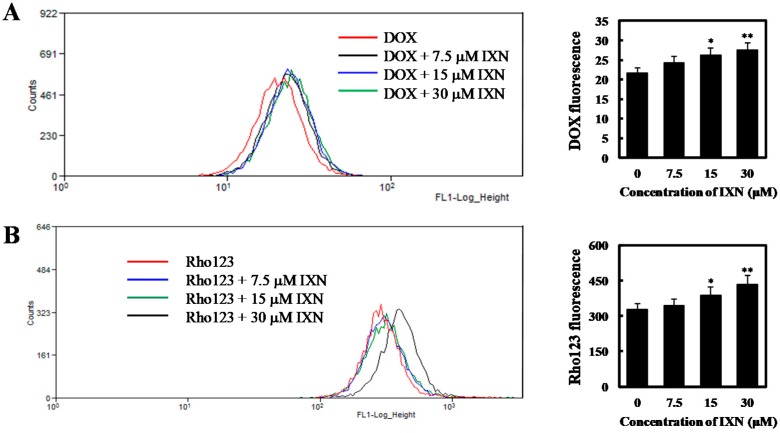

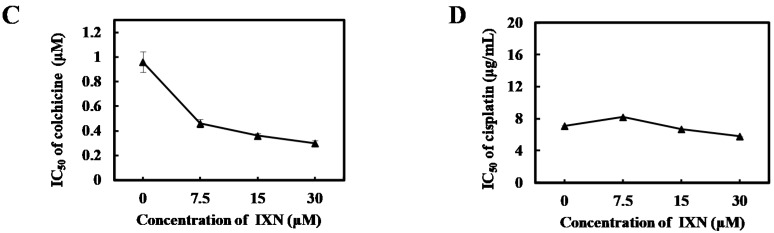
IXN inhibits the transporting function of ABCB1 in MCF-7/ADR cells. (**A**) Cells were incubated with DOX (3 μM) alone or in the presence of IXN (7.5–30 μM) for 24 h, then the autofluorescence of intracellular DOX in MCF-7/ADR was detected by flow cytometer moflo XDP (Beckman-Coulter). Histograms show the autofluorescence intensity of DOX. Values are expressed as means ± SD of three separate experiments (*p*-value relative to control group; * *p* < 0.05, ** *p* < 0.01). (**B**) MCF-7/ADR cells were pretreated with IXN for 6 h, and then with Rho123 for another 2 h. The intracellular Rho123 fluorescence intensity was analyzed by flow cytometry. Histograms show the mean fluorescence of Rho123. Values are expressed as means ± SD of three separate experiments (*p*-value relative to control group; * *p* < 0.05, ** *p* < 0.01). (**C**) IXN increases the sensitivity of MCF-7/ADR cells to ABCB1 substrate COL. (**D**) IXN does not enhance the sensitivity of MCF-7/ADR cells to cisplatin, which is not an ABCB1 substrate.

In order to investigate whether the observed synergism is related to intracellular accumulation of DOX, we first incubated MCF-7/ADR cells for 24 h with 3 μM DOX alone or in combination with 7.5 to 30 μM IXN, and measured the intracellular DOX using flow cytometry. We found that a relatively small amount of DOX accumulated in MCF-7/ADR cells. As a comparison, in the presence of IXN, much more DOX accumulated intracellularly in a concentration dependent manner (Figure 4A). We then evaluated the effect of IXN on ABCB1 transporting function by measuring the efflux of ABCB1 substrate Rho123. As shown in Figure 4B, IXN could enhance the intracellular Rho123 in a concentration dependent manner, indicating that IXN could block the efflux function of ABCB1. To further confirm IXN inhibition on ABCB1 transporting functions, we used ABCB1 chemotherapy substrate colchicine (COL) and assayed its cytotoxicity in MCF-7/ADR cells in the presence of IXN. Our results showed that IXN also sensitized MCF-7/ADR cells to COL (Figure 4C), however, it did not increase the sensitivity of MCF-7/ADR cells to cisplatin (Figure 4D), which is not an ABCB1 substrate. These results further confirmed that IXN modulated ABCB1 transporting function and showed synergism with DOX.

### 3.5. IXN Increased ABCB1 Expression and Stimulated the ATPase acTivity

**Figure 5:**
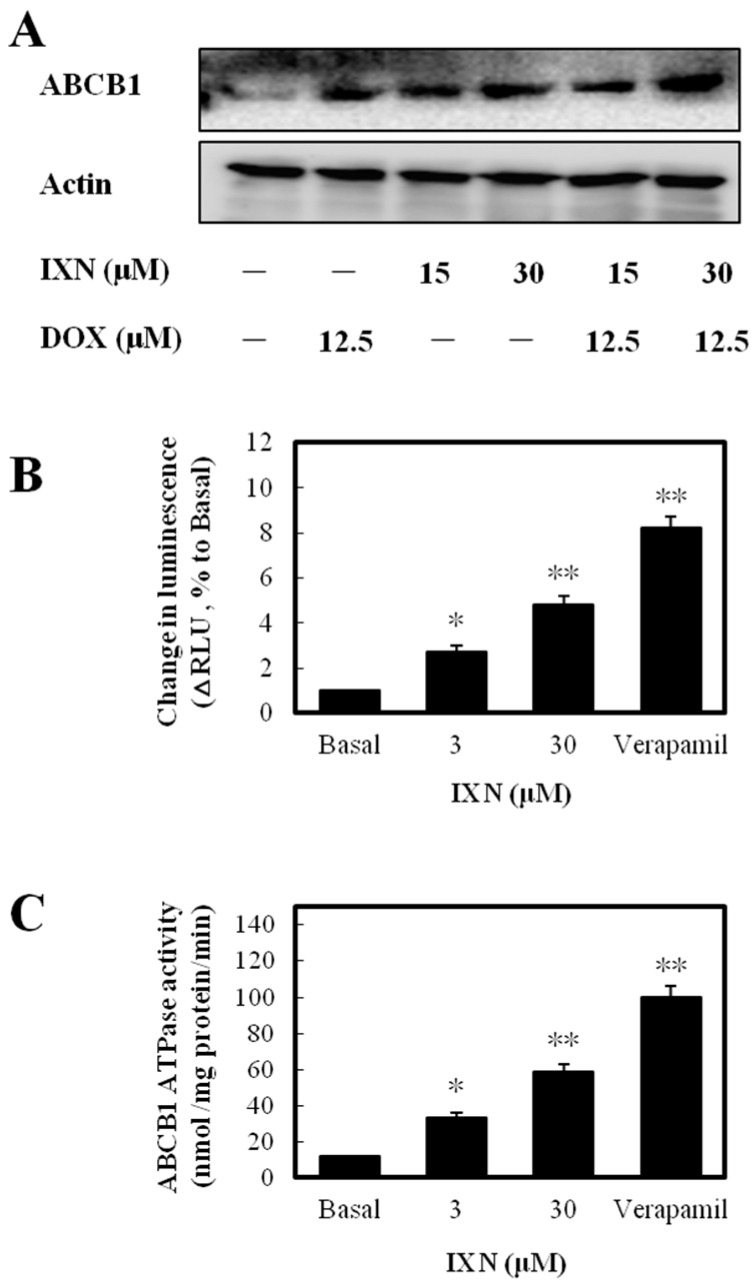
IXN increases ABCB1 level in MCF-7/ADR cells and is a substrate of ABCB1. (**A**) Increased expression of ABCB1 in MCF-7/ADR cells, after treatment with IXN (0–30 μM) alone or in the presence of DOX for 24 h. (**B**) IXN shows the ABCB1 substrate characteristics as the positive control verapamil. (**C**) IXN stimulates the ATPase activity of ABCB1. The ATPase activity of ABCB1 was determined as described in the materials and methods section.

To discover the molecular mechanism underlying ABCB1 function inhibition by IXN, we first detected the expression levels of ABCB1. After IXN treatment alone or in combination with DOX, the ABCB1 level was increased (Figure 5A). The increased expression level but inhibited transporting function clearly suggested that IXN affected ABCB1 ATPase activity. We next measured the IXN effect on ABCB1-mediated ATP hydrolysis and found that IXN, like the positive control verapamil, showed clearly ABCB1 substrate characteristics (Figure 5B) and stimulated ABCB1 ATPase activity (Figure 5C). These results suggested IXN acted as a competitive substrate of ABCB1 that blocked the DOX transportation.

### 3.6. IXN Binds to the Central TMD Site Where DOX Binds

To explore potential interactions between IXN and ABCB1, we constructed ABCB1 homology models and performed structural analysis and docking studies. Two human ABCB1 homology models were built based on the recently published mouse crystal structures which have two conformations with significant differences at the transmembrane helix-4 (TM4): an “open” conformation with a straight helical TM4, and a “closed” conformation with a kinked TM4 which partly closes the central TMD pocket of the transporter. Except the differences at the TM4, the rest of the parts of the two human ABCB1 models are essentially the same, including their nucleotide binding domains (NBDs).

**Figure 6:**
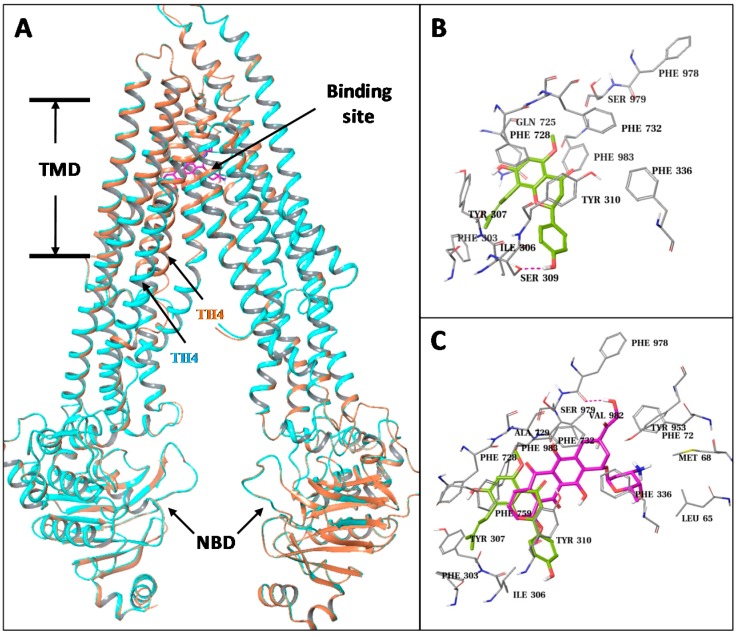
Structural presentation of the predicted ABCB1-ligands interaction. (**A**) The overlaid ABCB1 structural models. Models with straight helical TM4 and with kinked TM4 are represented in cyan-color and orange-colored ribbons, respectively. A docked ligand (DOX) at the transmembrane domain (TMD) site is shown in purple-colored solid sticks. (**B**) Close-up view of the docked result of IXN at the central TMD of ABCB1. IXN is shown in green-colored solid sticks. Binding site residues are shown in gray thin sticks. Hydrogen-bonds are shown in dashed lines. (**C**) Overlay of docked IXN and DOX. Results are presented as in (**A**,**B**).

We docked the IXN molecules into the central TMD pocket of each of the two models as well as to the ATP binding site at the NBD (Figure 6A). The resulting docking score at the central site of the closed model (−11.5/ kcal/mol) was significantly better than the open model (−9.7 kcal/mol) and the ATP binding site (−7.3 kcal/mol). The central TMD site is mainly formed by residues from TM4, TM5, TM6, TM7, and TM12. IXN only occupied a top portion that is close to TM5 (Tyr307, Phe303, Ile306, Ser309, and Tyr310) and TM7 (Gln725, Phe728) due to its relatively packed conformation (Figure 6B). As a comparison, we also docked DOX to the different binding sites of the two human ABCB1 models (Figure 6C). Similarly, the docked results of DOX favored the central TMD site of the closed model in comparison to the open model and the ATP site, with docking scores of −12.5 kcal/mol vs. −11.0 kcal/mol and −8.9 kcal/mol, respectively. While the DOX molecule also docked into the top region of the TMD site, it was closer to the side of TM1 (Leu65, Met68) and TM6 (Phe336) than TM5. Nonetheless, there are clear overlaps among the predicted binding sites of IXN and DOX (Figure 6C), indicating that IXN could competitively block the DOX binding to the transporter.

## 4. Discussion

IXN is the most abundant prenylflavonoid that exists in beer. It is beneficial to human health and showed multiple bioactivities [5]. Different from previous studies, the MCF-7/ADR cell line, which is resistant to many chemotherapeutic drugs, was used to explore the novel bioactivities of IXN. We, for the first time, revealed that IXN could reverse MCF-7/ADR resistance to DOX and the combined use of IXN with DOX showed a clear synergistic effect demonstrated by the enhanced effects on cell proliferation inhibition and apoptosis induction. This is consistent with the results of previous studies on XN, which is an analog compound of IXN and also a DOX sensitizer [13,19], suggesting that these prenylflavonoid compounds share the similar property of sensitizing DOX. These results provide additional information regarding the novel activities of IXN and imply its potential therapeutic applications.

ABCB1 plays a vital role in DOX resistance in MCF-7/ADR cells by stimulating DOX efflux. The observed synergistic effect with DOX led us to further explore whether IXN could modulate ABCB1 function(s). As expected, IXN significantly increased the intracellular accumulation of DOX and Rho123, both are ABCB1 substrates, which confirmed that IXN inhibited the efflux function of ABCB1. However, IXN treatment increased the expression level of ABCB1, which was possibly the feedback of IXN stimulation as an ABCB1 substrate. Such substrate-induced ABCB1 overexpression was also observed in intestinal epithelial cells [20]. Nonetheless, the exact mechanism(s) underlying the IXN-induced increased ABCB1 expression level need to be further investigated. It is possible that IXN reverses drug resistance by modulating the ATPase activity of ABCB1. Our results confirmed that IXN indeed acted as an ABCB1 substrate and stimulated the ABCB1 ATPase activity. Results of molecular docking studies showed that IXN could bind to ABCB1 at the central TMD site where DOX binds, indicating that IXN competitively blocked the DOX binding. It would be interesting to experimentally confirm the predicted IXN-ABCB1 interactions, such as through mutagenesis studies. Interestingly, previous studies have showed that IXN was also a substrate and inhibitor of ABCG2 [12], which is another drug efflux pump [2]. Therefore, modulation in other transporters, such as ABCG2, might also be involved in IXN-induced inhibition of DOX efflux. In the present studies, we revealed the prenylflavonoid compound IXN to be a substrate of ABCB1, which improves the understanding about the prenylflavonoids regarding their novel bioavailability in ABC-transporter-mediated drug interaction and drug resistance. This newly discovered synergistic effect with DOX suggests a possible application for overcoming ABC-transporter-mediated drug resistance in cancer chemotherapy.

## 5. Conclusions

In the present study, we revealed a novel bioactivity of prenylflavonoid IXN, namely, the synergistic effect with DOX on reversing ABCB1-mediated drug resistance in MCF-7/ADR cells. The synergistic effect was related to the inhibition of the ABCB1 efflux of DOX. Specifically, IXN bound to the DOX binding site and acted as a substrate and inhibitor of ABCB1 to inhibit DOX efflux. Our study, for the first time, demonstrated that IXN was a substrate of ABCB1 and could be used to overcome ABCB1-mediated multiple drug resistance.
